# Radiotherapy and palliative care outpatient clinic: a new healthcare integrated model in Italy

**DOI:** 10.1007/s00520-023-07584-y

**Published:** 2023-02-21

**Authors:** Romina Rossi, Flavia Foca, Luca Tontini, Martina Pieri, Simona Micheletti, Oriana Nanni, Mattia Altini, Ilaria Massa, Maria Caterina Pallotti, Marianna Ricci, Antonino Romeo, Maria Giustina Muolo, Gianluca Galeotti, Vanessa Valenti, Maria Valentina Tenti, Costanza Maria Donati, Maria Vittoria Pensieri, Alessio Giuseppe Morganti, Marco Maltoni

**Affiliations:** 1 Palliative Care Unit, IRCCS Istituto Romagnolo per lo Studio dei Tumori (IRST) “Dino Amadori”, Meldola, Italy; 2 Unit of Biostatistics and Clinical Trials, IRCCS Istituto Romagnolo per lo Studio dei Tumori (IRST) “Dino Amadori”, Meldola, Italy; 3 Radiotherapy Unit, IRCCS Istituto Romagnolo per lo Studio dei Tumori (IRST) “Dino Amadori”, Meldola, Italy; 4 Healthcare Administration, Azienda Unità Sanitaria Locale della Romagna, Ravenna, Italy; 5 Outcome Research, Healthcare Administration, IRCCS Istituto Romagnolo per lo Studio dei Tumori (IRST) “DinoAmadori”, Meldola, Italy; 6 Palliative Care Unit, Azienda Unità Sanitaria Locale della Romagna, Forlì, Italy; 7 Radiation Oncology, IRCCS Azienda Ospedaliero-Universitaria di Bologna, Bologna, Italy; 8 Radiation Oncology, Department of Experimental, Diagnostic and Specialty Medicine, University of Bologna, Bologna, Italy; 9 Medical Oncology, “San Salvatore” Hospital, Univerity of L’Aquila, L’Aquila, Italy; 10 Department of Biotechnological and Applied Clinical Sciences, Univerity of L’Aquila, L’Aquila, Italy; 11 Medical Oncology Unit, Department of Specialized, Experimental and Diagnostic Medicine (DIMES), University of Bologna, Bologna, Italy

**Keywords:** Palliative care, Palliative radiotherapy, Symptom management, Aggressiveness of care

## Abstract

**Background:**

On the basis of substantial evidence demonstrate that palliative care combined with standard care improves patient, caregiver, and society outcomes, we have developed a new healthcare model called radiotherapy and palliative care (RaP) outpatient clinic were a radiation oncologist and a palliative care physician make a joint evaluation of advanced cancer patients.

**Methods:**

We performed a monocentric observational cohort study on advanced cancer patients referred for evaluation at the RaP outpatient clinic. Measures of quality of care were carried out.

**Results:**

Between April 2016 and April 2018, 287 joint evaluations were performed and 260 patients were evaluated. The primary tumor was lung in 31.9% of cases. One hundred fifty (52.3%) evaluations resulted in an indication for palliative radiotherapy treatment. In 57.6% of cases was used a single dose fraction of radiotherapy (8 Gy). All the irradiated cohort completed the palliative radiotherapy treatment. An 8% of irradiated patients received the palliative radiotherapy treatment in the last 30 days of life. A total of 80% of RaP patients received palliative care assistance until the end of life.

**Conclusion:**

At the first descriptive analysis, the radiotherapy and palliative care model seem to respond to the need of multidisciplinary approach in order to obtain an improvement on quality of care for advanced cancer patients**.**

## Introduction

Radiotherapy (RT) is one of the principal oncological treatments, and about half of all RT have been delivered with a palliative intent to control or prevent cancer symptoms [1]. With the improvements in cancer care and the development of high precision technology, the distinction between curative RT and palliative RT (PRT) is becoming blurred, and the therapeutic decision in PRT is becoming particularly complex [2]. Moreover, it is to be considered that in PRT, the therapeutic decision concerns not only whether to do or not do the treatment, but also the choice of the appropriate fractionation (single dose, hypofractionation, etc.), the correct timing of RT treatment to ensure patients the symptoms relief or their prevention, as well as the best RT technique to use (3DRT, VMAT, tomotherapy, etc.).

Another difficult task is prognostication. It is known that survival prognostication by physicians is difficult to establish [3]. Subsequently, the PRT over-treatment or non-beneficial treatment for advanced cancer patients’ risks is increasing [4–6].

A systematic review showed that the overall PRT utilization rates in the last 30 days of life were in the range between 5 and 10% among patients who died of cancer and 9–15.3% among patients receiving PRT who died of cancer [7]. Most patients received ten fractions of RT and the single fraction of RT varied from 0 to 59%. So, there was a high rate (53–83%) of incomplete treatment in the patient population with multifraction courses [7].

Regarding this matter in our retrospective experience, the proportion of patients receiving RT in the last 30 days of life was similar to that of other studies, but we had a higher proportion of patients who underwent schedules of 2–10 fractions or more than ten fractions of RT. In this study, population more than 40% are the interrupted courses and the planned but never started courses for rapidly worsening clinical conditions. We also looked deeper at the costs of this cohort, and we observed that around 7.7% and 30.3% of the total cost was associated with patients who never started RT or who discontinued RT. The amount of resources used for non-beneficial treatments in our population indicated that careful patient selection and more accurate survival prognostication are key to reducing the risk of inappropriate therapies and costs [8].

For Lutz et al., the crux of the matter in the modern PRT is to identify the RT intent, and these authors suggested that the promising advances in RT must be applied while keeping general PC approaches in mind [1].

During the past 25 years, various PRT models have been developed to optimize the outcomes of PRT, since this issue has important clinical and policy implications.

In 1996, in Toronto, an outpatient palliative care clinic called Rapid Response Radiotherapy Program (RRRP) started which had the aim to respond to the growing problem of a long waiting time for PRT and has evolved into an academic, patient-centered for symptom relief of advanced cancer patients [9]. This efficient care model has been exported in other RT units in Canada, the USA, and Australia. Each RRRP experience improved the program by incorporating the service of other oncology-related professionals, building a multidisciplinary, and incorporating of advanced RT technologies [10, 11].

In order to meet the clinical needs of patients with bone metastases who underwent PRT, a multidisciplinary experience with dedicated ambulatory assessment was developed into the RRRP. Weekly, a team composed of a radiation oncologist, a registered nurse, a nurse practitioner, a pharmacist, a radiation therapist, an occupational therapist, a social worker, and registered dietician evaluated the patients using validated-specific screening tools. All the personnel had palliative care and/or supportive care training. The analysis of this multidisciplinary assessment proved the feasibility of the model and revealed positive finding for decreased symptom distress in the study population [12, 13].

In Italy since 2012, the Italian Association of Radiation oncology (AIRO) recommended the need for prospective long clinical trials to define the clinical rationale of advanced technologies and to identify potentially clinically effective use [14]. In the same period, the Italian Association of Radiation Oncologist (AIRO) and Slow Medicine published a paper on the five practices at risk of inappropriateness and suggested “Not recommended the use of special techniques without a reasoned opinion from the oncologist radiotherapist” [15].

Meanwhile, PRT trials were conducted, seminal authors demonstrated that palliative care (PC) combined with standard care improves patient, caregiver, and society outcomes [16, 17].

Considering the substantial evidence, it has been demonstrated that in PRT, the multidisciplinary approach is feasible and effective, and that PC combined with standard care impact on quality of care reducing aggressiveness of care near death [18, 19], we have developed a new integrated healthcare model between radiotherapy and palliative care unit, called the radiotherapy and palliative care (RaP) outpatient clinic, which is the first in Italy to the best of our knowledge.

This paper, after examining the background and the rationale, will go into detail about the framework and the results of the first 2 years of the new integrated healthcare model. In particular, this first descriptive analysis will examine the outcomes of the RaP outpatient clinic in terms of indication to PRT, the PRT characteristics (doses, interrupted treatment), use of PRT at the end of life, and the place of death [20, 21].

## Materials and methods

### Study design and study objective

In our single, tertiary oncologic academic institution, we performed an observational cohort study on advanced cancer patients referred for evaluation at the RaP outpatient clinic. In the RaP outpatient clinic entered patients aged more than 18, with multimetastatic or locally advanced cancer, any histology. Hematological patients are enrolled too, while the oligo-progression cancer patients were excluded. The patients were referred from the Medical Oncology or Hematological Unit of our cancer center.

The primary aim of RaP outpatient clinic is to improve the appropriateness use of PRT in advanced cancer patients and to refer them to palliative care settings in a timely manner by a multidisciplinary approach. So in the first analysis of RaP outcomes, quality of care measures were used such as short PRT fractionation, interrupted PRT, use of RT in the last 30 days of life treatment, and place of death in palliative care services.

### Radiotherapy and palliative care outpatient clinic model

In this integrated healthcare model, a dedicated team of radiation oncologists and palliative care specialists makes a joint evaluation of advanced cancer patients referred from the Medical Oncology Unit. The dedicated clinical team is supported from a nurse of Rt Unit. The RaP visits were explained one morning, a week in the radiotherapy consulting room. When the patients arrive, the nurse performs the triage and assessment using ESAS scales and EORTC15Pal questionnaire and communicates with the RaP clinicians before they start the visit. In the meantime, the two physicians study the clinical history and the diagnostic imaging; after that, the two physicians make a joint clinical examination of the patient, using systematically clinical and prognostic indicators, as Performance status and Pap Score. Finally, on the basis of all data collected, they have a joint discussion; and they make a joint decision, which involves the indication for PRT, the optimization of pharmacological pain treatment, and referral to the appropriate palliative care setting, which was necessary.

The non indication for PRT is made considering both the clinical condition of the patient (e.g., very short prognosis, deterioration of clinical condition) and the characteristics for PRT target lesions (site, number, bone critical lesion).

A follow-up visit is planned at 1 month after receiving PRT if indicated or 1 month after the first RaP visit if RT treatment is not indicated.

During the follow-up visit, as in the first Rap visit, the clinical assessment is done using ESAS scales and EORTC15Pal, Performance status, and the EORTC toxicity scale in patients underwent to PRT. All date were collected in a dedicate database.

### Statistical analysis

Frequencies were calculated for categorical variables. For continuous variables, median (min–max) was shown. Overall survival (OS) time was calculated from the date of therapy initiation until the last follow-up visit or death. All statistical analyses were performed using Stata/SE version 15.1 for Windows (StataCorpLP, College Station, TX, USA).

## Results

Between April 2016 and April 2018, 287 joint evaluations were carried out in the RaP outpatient clinic on 260 patients.

One hundred forty (53.8%) were male, the median age was 69.2 years (range: 36.9–93.5), and primary tumor was lung in 31.9% of cases as shown in Table 1. 142/260 patients (49%) attended a RaP follow-up visit. One hundred fifty (52.3%) evaluations resulted in an indication of PRT treatment, while in 137 cases (47.7%), PRT treatment was not indicated at the first rap visit.

**Table 1:** Characteristics of the 260 patients involved in the study

Characteristics	*N* (%)
Median age (range)	69.2 (36.9–93.5)
Sex	
Male	140 (53.9)
Female	120 (46.1)
Site of disease	
Lung	83 (31.9)
Breast	48 (18.5)
Prostate	29 (11.2)
Gastro-intestinal	23 (8.9)
Melanoma	15 (5.8)
Bladder	13 (5.0)
Kidney	12 (4.6)
Others	37 (14.1)

The 140 patients were irradiated on 170 lesions, and 27(19%) patients were irradiated in more than one site. The bone and brain were the most frequent irradiated site, 71% and 14% respectively; more details are showed in Table 2.

**Table 2:** RT characteristics

Characteristics (site of irradiation)	Lesion *N* = 170 (%)
Column	63 (37.1)
Bone	58 (34.1)
Brain	22 (12.9)
Abdomen nodes	8 (4.7)
Pelvic lesion	7 (4.1)
Lung lesion	4 (2.4)
Lateral-cervical	4 (2.4)
Others	4 (2.4)
Single dose	
8 Gy	98 (57.6)
4 Gy	36 (21.2)
5 Gy	23 (13.5)
6 Gy	6 (3.5)
3 Gy	6 (3.5)
10 Gy	1 (0.7)
Total dose	
8 Gy	98 (57.6)
20 Gy	36 (21.2)
25 Gy	19 (11.2)
18 Gy	6 (3.5)
15 Gy	4 (2.4)
30 Gy	7 (4.1)
Fractionation	
1	99 (58.2)
5	53 (31.2%)
3	12 (7.1%)
10	6 (3.5%)
RT technique	
VMAT	79 (46.5)
TOMO	38 (22.4)
3D	53 (31.1)

Three patients did not undergo a TC due to worsening conditions, such as the occurrence of an acute event or claustrophobia. After TC, other eight patients did not undergo RT due to worsening clinical conditions.

Considering the RT characteristics, we observed that the majority fractionation use was a single dose and no more than ten fractions were administered (Table 2).

At the time of analysis, 103/260 patients were alive, 142 had died, and 15 were unknown.

**Fig. 1:**
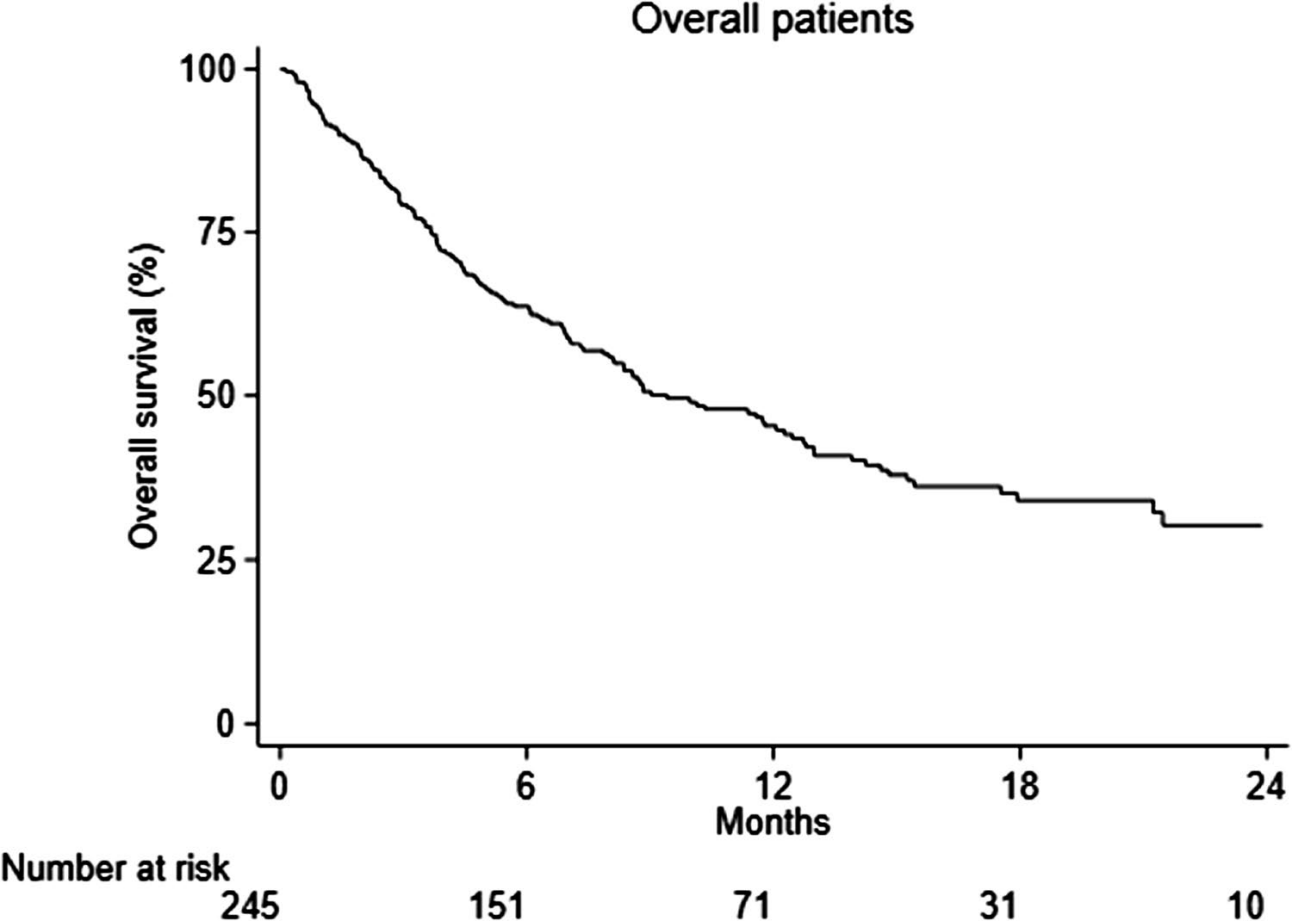
Survival of RaP patients

**Fig. 2:**
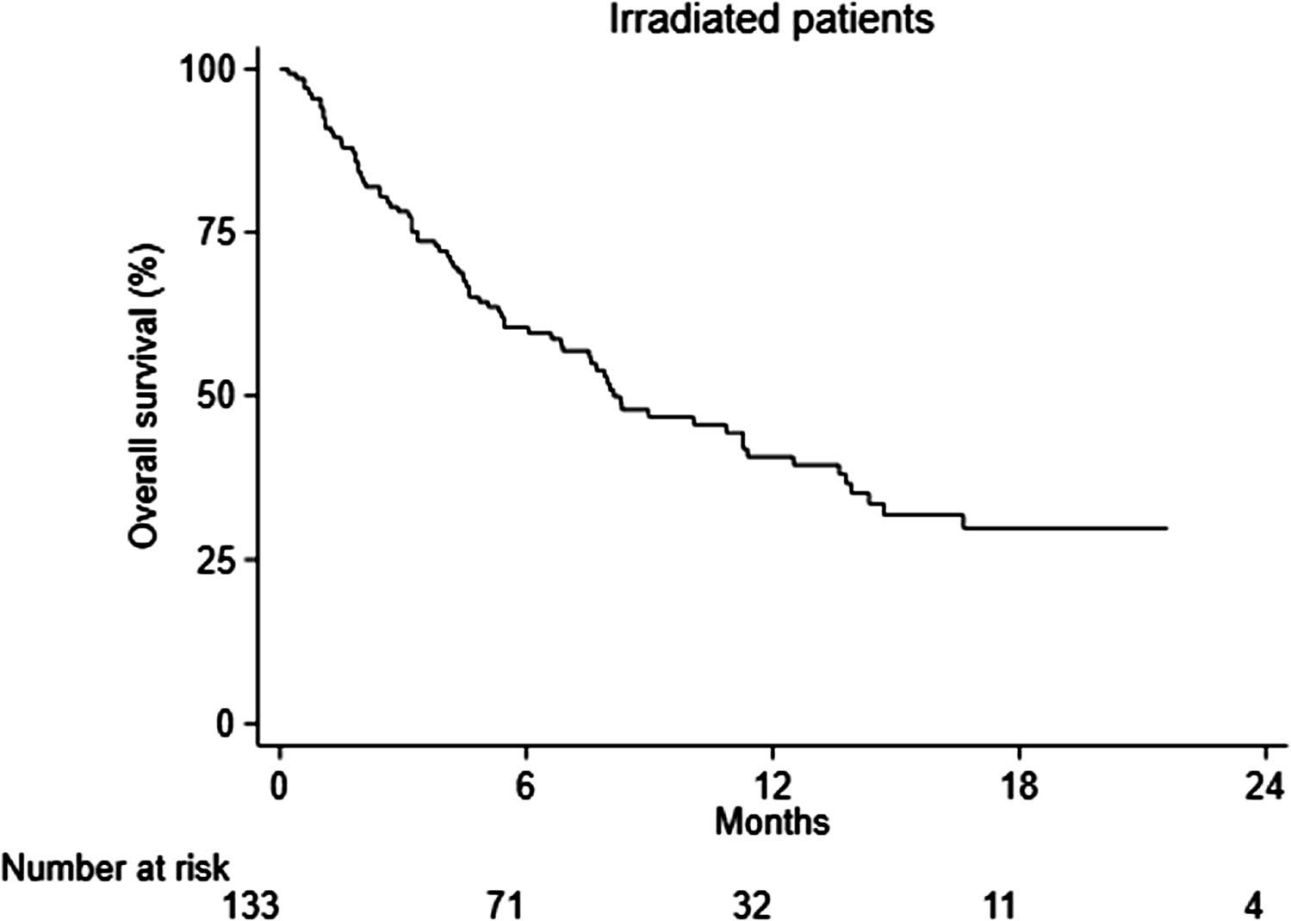
Survival of irradiated patients

Total median survival of all analyzed patients was 9.4 months (95% CI: 7.9–12.7 months) while for the irradiated patients subgroup, median survival was 8.1 months (95% CI: 6.6–11.4 months) (Figs. 1 and 2 respectively).

Considering the place of death, 122 patients (85.9%) were evaluable: of these, 76 (62.3%) had died in a hospice, 20 (16.4%) in a home care setting, and 26 (21.3%) in an acute unit.

A total of 80% of RaP patients received palliative care assistance until the end of life. All clinical characteristics of the patients who participated in the first 2 years of RaP outpatient clinic will be published in a dedicated article. Seventy-seven irradiated patients had died and all completed the PRT treatment as planned. Six (8%) patients received PRT in the last 30 days of life. Of this subset of patients, 5 (83%) died in a palliative care setting, 4 at hospice, and 1 in home-care respectively; only one patient died in hospital for an acute event. Four (67%) patients received a single dose of PRT in the last 30 days of life; two patients underwent five fractions and completed it before dying.

## Discussion

In the era of precision medicine, ensuring the better quality of care in oncology remains a challenging question to answer. Over the past decade, multiple randomized controlled trials have demonstrated that timely involvement in specialist palliative care concurrent with oncological care can improve health outcomes, quality of care, and end-of-life care [22]. The strong evidence by Temel randomized clinical trials where integration of palliative care in oncology resulted in better illness understanding than oncologic care alone [16].

In addition, several initiatives highlighted the need of integration between palliative medicine and radiotherapy. Yet, these efforts have remained largely independent, without attention to overlap; and integration and synergies between radiotherapy, palliative medicine, and other global health initiatives deemed necessary and will be essential in bringing palliative radiotherapy to patients around the globe [23].

Given the substantial evidence, at our institute, we have developed a new integrated healthcare model, the “**R**adiotherapy **a**nd **P**alliative care (RaP) outpatient clinic,” where palliative care was integrated with radiotherapy; the present paper showed the first analysis focused on some quality of care measures.

The “backbone” of RaP outpatient clinic model is the multidisciplinary approach; this model permits the so-called “integrated decision-making approach” claimed as shared decision-treatment between radiotherapists and palliative care specialists to direct patient care and to direct prognostication and real needs. In fact, the characteristic of RaP outpatient clinic is the joint and simultaneous evaluation of cancer patients by radiation oncologist and palliative care physician.

The interaction between the two physicians as well as the collaboration of dedicated nurses and the systematic use of clinical scales allows a more careful assessment of patient characteristics and a better prognostic evaluation of life expectancy and guarantees a better management of supportive care and RT acute-adverse events. The resulting ability to tailor palliative treatment as what has just been defined is essential to make healthcare decisions to avoid unnecessary PRT treatment, allowing a timely referral to supportive and PC, when necessary, and finally for a personalization of end-of-life care as a commission of expert recommended [24].

This is probably the way to read our result where 47.7% of cases was PRT treatment was not indicated at the first rap visit. We believe that a proper balance between prognostication, clinical condition, and RT treatment to administer can enhance the final quality of life outcomes.

Moreover, with the multidisciplinary approach as well as the “no PRT treatment” decision, the patient remains in charge of the palliative care which could permit the patient and family to feel taken care of.

From the healthcare administration point of view, the RaP model could improve use of RT services in terms of equity of access and reducing waiting times. In fact, a greater selection of patients to be irradiated allows a consequent re-allocation of RT treatment slots not used, with a possible positive impact on waiting time for PRT.

The Rap model would guarantee a better continuity of care with more adequate use of palliative care services at the end of life, and we observed 80% of patients die in palliative care settings.

In the Rap outpatient clinic, the majority of irradiated lesions were bone metastases, as expected in PRT. In our irradiated cohort, short fractionation was the most used, in accordance with the stronger recommendation in the matter, in particular the single-dose (8 Gy) was used in 57.6% of irradiated targets [25].

Considering the use of PRT in the last 30 days of life, a seminal systematic review by Park et al. revealed a range from 5 to 10% among patients who died from cancer and from 9 to 15.3% among those receiving PRT [26]. A more recent survey by Wu et al. showed 24% of patients received PRT within 30 days of life, and 42% of the irradiated patients did not complete their planned RT course [27].

In our center before Rap started a previous retrospective analysis on PRT showed a proportion of patients undergoing RT in the last 30 days of life similar to the other studies, although a higher proportion of our patients underwent a 2–10 fraction and > 10 fraction RT. And more than 40% of the study population was the interrupted courses and the planned but never started courses. In our current study, the percentage of irradiated patients in the last 30 days of life was only 8%, and into the entire irradiated cohort, no interrupted PRT treatment was observed [7, 8].

A power of RaP outpatient clinic model consists in the training opportunity in palliative care for radiotherapy personnel as well as in PRT for the palliative care physician. In fact, many radiation oncologists as well as nurses had no formal education in hospice and palliative care during their medical school training and residency [28].

A recent survey documented that there was also a knowledge gap in PRT among palliative care physician, so we believe that the Rap model permits an overlapping between the two disciplines, which could help overcome barriers to obtain high-quality PRT into high quality PC [29].

Finally, the close interaction with palliative medicine supports the RT effort in research to answer the many open questions.

Our study is limited by its observational nature and small sample size that limited our ability to detect further interactions between groups. These preliminary and promising results lead us to study the model with a stronger method and also to deepen many of the aspects that emerged from this first step.

## Conclusions

Substantial evidence demonstrated that a multidisciplinary approach is preferable for improving quality of care and quality of life in advanced cancer patients. Looking forward in this direction, we developed a new healthcare integrated model between radiotherapy and palliative care for advanced cancer patients, the first in Italy to the best of our knowledge. The first analysis of RaP outpatient clinic activity showed that this integrated model facilitates the decision-making process on PRT, could reduce the risk of unnecessary PRT, and allowing timely referral to supportive and palliative care services. To assess the impact of this integrated model on the promising outcomes, we are going to perform a randomized study of RaP outpatient clinic versus standard PRT evaluation alone.

## Data Availability

The datasets generated and/or analyzed during the current study are available from the corresponding author on reasonable request.
